# Central Texas Medical Society: Programme

**Published:** 1892-02

**Authors:** W. R. Blailock, W. O. Wilkes

**Affiliations:** Pres.; Sec’y.


Programme for next meeting of the Central Texas Medical Association, Tuesday April 12, 1892:

1 "Diphtheria,—Etiology and Treatment," Dr. R. C. Nettles; discussion by Drs. H. C. Ghent and W. T. Baird.

2. "Prolapse of Uterus,—Its Management," Dr. J. M. Frazier; discussion by Drs. J. W. Hunter and J. H. Sears.

3. "Tertiary Syphilis,—Its Ravages in Nerve, Muscle and Bone Tissue," Dr. O. I. Halburt; discussion by Drs. A. H. Snead and W. E. Menefee.

4. "Prolapsus Ani,—Its Management," Dr. Douglas Harris; discussion by Drs. W. W. Greer and J. D. Law.

5. "Otorrhœa in Children,"—Dr. J. R. Ferrell; discussion by Drs. A. M. Curtis and M. T. McNeill.

Reports and papers invited.

W. R. BLAILOCK, M. D., Pres.
W. O. WILKES, M. D., Sec'y.

**Cheatham's Treatment for Trachoma.**—Dr. Cheatham, in a lecture to his class in the Medical Department University, of Louisville, *Ophthalmic Record*, August, 1891, says:

"In the management of this most obstinate disease, I use atropia sulph., in the saturated solution of acid boracic, from the beginning to the end. It serves several purposes for me; it puts the eyes at rest, it is antiseptic, it strangulates some of the trachomatous bodies, and the hypertrophied papillæ; it relieves corneal photophobia. The boracic acid in the solution also prevents local irritation, which sometimes occurs from the long continued use of atropia sulph. I frequently add to an ounce of the solution, zinc sulph. gr. $\frac{1}{4}$ or $\frac{1}{8}$ for the same purpose. When a case of trachoma presents itself to me, as I stated before, I order the use of the atropia solution immediately, for the purpose I have given, and also to get the refraction of the eye. In all stages of this disease, it is my aim at the earliest moment to get the correct refraction; I cannot recall a case now in which I failed to find an error. To this error of refraction I attribute the primary cause of the disease; the disease I consider a specific one; the error of refraction simply prepares the soil for the reception of the germ; correct this error as soon as possible, and we prevent many relapses of the disease. So I repeat, it is my constant endeavor to find the existing error, and correct it by glasses."

After correcting the error of refraction, Dr. Cheatham orders hyd. oxid. flav. gr. viii, to xii, valeline ℥i, also cupri. sulph. in crystal or solution; one to be applied to the granulated lid for two weeks, then the other for two weeks, alternating. Afterwards bathe the eye in hot water ten or fifteen minutes. To be used not nearer than three hours to bedtime. Most acute cases yield to this treatment.

But it is in the severe cases he uses the curette or scarification; he does not mention compression, or expression in this lecture.

He has given up infusion of jequirity, and uses an impalpable powder; to this he ascribes wonderful results, even in cases before intractable. The Doctor is so impressed with powdered jequirity that he says:

"To those who are afraid to be as bold as I am in its use, I suggest they use it on their more hopeless cases. There are many people, in this country, who are considered hopelessly blind, to whom useful vision can be restored by the use of jequirity powder."

A pretty broad assertion.

